# Risk Factors and Outcomes of Pediatric Poisoning: A Cross-Sectional Study From a Tertiary Care Hospital in Peshawar, Pakistan

**DOI:** 10.7759/cureus.83463

**Published:** 2025-05-04

**Authors:** Huma Gul, Nasar Rashid, Mustafa Kamal, Asad Khan, Maaz Ahmad, Israr Hussain

**Affiliations:** 1 Pediatrics and Child Health, Hayatabad Medical Complex, Peshawar, PAK; 2 Pediatric Critical Care, Royal Hospital for Children and Young, Edinburgh, GBR

**Keywords:** children, medications, organophosphates, outcomes, pediatrics, petroleum, poisoning, risk factors

## Abstract

Background: Pediatric poisoning is a common and potentially life-threatening issue in lower-middle-income countries like Pakistan. It often goes unnoticed in households with young children and accounts for a substantial number of emergency department (ED) visits.

Objective: This study aims to identify the risk factors and common agents of acute poisoning in children and to determine their outcomes.

Materials and methods: In this cross-sectional study, the records of children under 14 years of age who presented to the pediatric ED with a history of poisoning within the preceding 48 hours were reviewed. The study period spanned from January 1, 2024, to December 31, 2024. A total of 154 patients were included. Data recorded included age, gender, poisoning agent, route, mode, and place of poisoning; whether the child was hospitalized or discharged after observation; length of stay; intensive care unit (ICU) admission; need for organ support; and hospitalization outcomes. All analyses were performed using SPSS Statistics version 21 (IBM Corp. Released 2012. IBM SPSS Statistics for Windows, Version 21.0. Armonk, NY: IBM Corp.). Risk factors for adverse outcomes were assessed using the Chi-square test or Fisher’s exact test, as appropriate. A p-value below 0.05 was considered statistically significant.

Results: Of the 154 patients, the majority (74.7%, n = 115) were under the age of three years, and most were hospitalized (88.3%, n = 136). All poisoning cases involved accidental ingestion, with the majority occurring at home (96.1%, n = 148). Organophosphorus compounds were the most common poisoning agents (27.9%, n = 43), followed by medications (26.6%, n = 41) and petroleum products (17.5%, n = 27). Most patients (80.9%, n = 110) remained hospitalized for up to three days, with a mean duration of stay of 2.8 ± 1.8 days. The majority of hospitalized patients survived (87.5%, n = 119). Although length of stay, ICU admission, and need for organ support were significantly associated with mortality in univariate analysis, none were significant predictors of mortality in multivariate analysis: length of stay (AOR: 1.54; 95% CI: 0.27-8.86; p = 0.631), ICU admission (AOR: 0.967; 95% CI: 0.05-18.64; p = 0.982), and organ support (AOR: 14.04; 95% CI: 0.55-358.32; p = 0.110).

Conclusions: Pediatric poisoning predominantly affects children under the age of three years, with most incidents occurring at home. Organophosphates, medications, and petroleum products were the most commonly involved agents. High rates of hospitalization and mortality highlight the severity of the issue. To mitigate this problem, there is a pressing need for parental education, childproofing measures at home, and improved healthcare infrastructure to prevent poisoning incidents and enhance patient outcomes.

## Introduction

Pediatric poisoning is a common and potentially life-threatening issue that often goes unnoticed in households with young children. It is responsible for a substantial number of ED visits. It was reported as the third most common cause of unintentional injuries in children under 15 years of age. Children under the age of 20 years comprised 13% of the deaths resulting from accidental poisoning. Compared to the developed world, the mortality of children from poisoning is higher in developing countries [1]. In addition, there may be long-term consequences of pediatric poisoning incidents, including potential neurological damage or developmental delays that may result from exposure to toxic substances at a young age [2].

The majority of the children presenting to hospitals with acute poisoning are younger than five years of age, and most cases are accidental [3-5]. The high incidence in this age group is due to their inherent inquisitiveness, higher oral exploratory activity, newly acquired mobility, and hand skills. Male children are frequently involved in acute poisoning, especially in the younger age groups. However, there is a shift to female predominance during adolescence [6,7]. There is regional variation in the agents involved in pediatric poisoning. In 2023, Gaw et al. reported opioids and over-the-counter medications as the most common agents of fatal poisoning in young children in the United States [8]. In underdeveloped nations, petroleum-based products like kerosene and organophosphorus compounds are the most frequently ingested during accidental poisoning [9,10]. Most childhood poisoning incidents take place at home. In a study from Southwest China, 90.4% of poisoning cases occurred at home [11]. Similarly, in Bangladesh, about 82% of poisoning incidents happened in the parents' homes, often in the bedroom [12]. These findings suggest that most of these events result from inadequate storage of medications and household chemicals and are thus preventable. The mean duration of hospitalization has ranged from 1.8 to 4.8 days and is influenced by the type of poison, the severity at presentation, and the interventions required [13,14]. The overall mortality of poisoning in children is generally low, but the reported figures vary by region and by the agents of poisoning. Mortality rates of 0.3% to 8.7% have been reported from different regions [10,15,16].

Pediatric poisoning is a significant public health issue in lower-middle-income countries like Pakistan. The widespread availability of toxic substances, their inadequate storage, and poor supervision of children due to insufficient parental knowledge contribute to the issue [1]. Given the paucity of recent data on pediatric poisoning from Pakistan, this study aims to identify the common agents of poisoning in children, explore the factors contributing to pediatric poisoning cases, and determine their outcomes. The results will help raise awareness among healthcare workers and will influence policymakers to propose potential prevention interventions and outreach programs to increase parental awareness. This will be important in resource-limited settings where access to antidotes or specialized care is limited.

## Materials and methods

This cross-sectional study was carried out in the pediatrics department of Hayatabad Medical Complex, Peshawar, Pakistan. Ethical approval was obtained from the Hospital Research and Ethical Committee of Hayatabad Medical Complex (approval number: 2449). Records of children under 14 years who presented to the pediatric ED with a history of poisoning in the preceding 48 hours were reviewed and included in the study. Patients hospitalized in any other health facility before presenting to Hayatabad Medical Complex and those with incomplete records were excluded. The study period ranged from January 1, 2024, to December 31, 2024. A total of 154 patients met the eligibility criteria during the study period and were included.

A structured questionnaire was used to collect the following information: age, gender, the agent of poisoning, the route of poisoning (oral, inhalational, transdermal), the mode of poisoning (accidental, homicidal, suicidal), the place of poisoning (home, outside home), hospitalized or discharged from the ED after observation, the duration of hospitalization (days), intensive care admission, the need for organ support (ventilation, dialysis, ionotropic support), and the outcome of hospitalization (survived, not survived).

Data were analyzed using SPSS Statistics version 21 (IBM Corp. Released 2012. IBM SPSS Statistics for Windows, Version 21.0. Armonk, NY: IBM Corp.). Descriptive statistics (mean ± standard deviation; frequency and percentage) were calculated for the above-mentioned variables. The risk factors were compared between the survivors and non-survivors, and the statistical significance of any differences was determined using the Chi-square test/Fisher’s exact test. A p-value below 0.05 was taken as significant for all analyses.

## Results

The mean age of the study participants was 2.5 ± 1.5 years, and 74.7% (n = 115) were under the age of three years. Boys (57.1%, n = 88) outnumbered girls. Out of 154 patients, 136 (88.3%) were hospitalized. The mean length of stay was 2.8 ± 1.8 days, and 80.9% (n = 110) of patients remained hospitalized for up to three days. All poisonings were accidental ingestions, and the majority took place at home (96.1%, n = 148). Organophosphates were the most common agents of poisoning (27.9%, n = 43). Most hospitalized patients survived (87.5%, n = 119). The characteristics of the study population are summarized in Table 1.

**Table 1:** Characteristics of the study participants (n = 154) * data for hospitalized cases (n = 136) ED: emergency department

Variables	No. (%)
Age groups	
Up to 3 years	115 (74.7%)
Older than 3 years	39 (25.3%)
Length of stay*	
Up to three days	110 (80.9%)
More than three days	26 (19.1%)
Gender	
Male	88 (57.1%)
Female	66 (42.9%)
Agent of poisoning	
Organophosphates	43 (27.9%)
Medications	41 (26.6%)
Petroleum products	27 (17.5%)
Opioids	15 (9.7%)
Rat poison	10 (6.5%)
Poisonous plants	8 (5.2%)
Detergents	7 (4.5%)
Others	3 (1.9%)
Place of poisoning	
Home	148 (96.1%)
Outside home	06 (3.9%)
Triage	
Hospitalized	136 (88.3%)
Discharged from ED	18 (11.7%)
Intensive care admission*	
No	128 (94.1%)
Yes	08 (5.9%)
Organ support*	
No	118 (86.8%)
Yes	18 (13.4%)
Mechanical ventilation*	
No	130 (95.6%)
Yes	06 (4.4%)
Ionotropic support*	
No	124 (91.2%)
Yes	12 (8.8%)
Dialysis*	
No	132 (97.1%)
Yes	04 (2.9%)
Outcome*	
Survivors	119 (87.5%)
Non-survivors	17 (12.5%)

Older children had a higher mortality rate compared to those younger than three years (10.8% (n = 12) vs 20% (n = 05)), though this difference was not statistically significant (p = 0.311). Univariate analysis showed that a length of stay exceeding three days (p = 0.021), the requirement for intensive care admission (p = 0.001), mechanical ventilation (p = 0.026), ionotropic support (p < 0.001), or dialysis (p = 0.006) were significantly associated with poorer outcomes, specifically non-survival. Table 2 provides a summary of the association between various risk factors and patient outcomes for those hospitalized due to acute poisoning.

**Table 2:** Relationship between various risk factors and survival outcomes in pediatric patients admitted for acute poisoning (n = 136) # Fisher’s exact test, $ Chi-square test, * data for the three most frequent agents of poisoning

Variables		Outcome, no. (%)		p-value
		Survivors (n = 119)	Non-survivors (n = 17)	
Age groups	Up to 3 years	99 (89.2%)	12 (10.8%)	0.311^#^
	Older than 3 years	20 (80%)	05 (20%)	
Length of stay	Up to three days	100 (90.9%)	10 (9.1%)	0.021^#^
	More than three days	19 (73.1%)	07 (26.9%)	
Gender	Female	55 (88.7%)	07 (11.3%)	0.696^$^
	Male	64 (86.5%)	10 (13.5%)	
Agent of poisoning* (n = 99)	Organophosphates	34 (87.2%)	05 (12.8%)	1.000^#^
	Medications	29 (87.9%)	04 (12.1%)	
	Petroleum products	23 (85.2%)	04 (14.8%)	
Place of poisoning	Home	116 (87.9%)	16 (12.1%)	0.418^#^
	Outside home	03 (75%)	01 (25%)	
Intensive care admission	No	116 (90.6%)	12 (9.4%)	0.001^#^
	Yes	03 (37.5%)	05 (62.5%)	
Organ support	No	111 (94.1%)	07 (5.9%)	<0.001^#^
	Yes	08 (44.4%)	10 (55.6%)	
Mechanical ventilation	No	115 (89.1%)	14 (10.9%)	0.026^#^
	Yes	03 (50%)	03 (50%)	
Ionotropic support	No	114 (91.9%)	10 (8.1%)	<0.001^#^
	Yes	05 (41.7%)	07 (58.3%)	
Dialysis	No	118 (89.4%)	14 (10.6%)	0.006^#^
	Yes	01 (25%)	03 (75%)	

When adjusted, the risk factors identified as significant in univariate analysis did not maintain their statistical significance in multivariate logistic regression analysis. This indicates that the observed associations in the univariate analysis were likely attributable to confounding effects or the relatively smaller sample of non-survival cases (Table 3).

**Table 3:** Multivariate logistic regression analysis for adjusted odds of non-survival * reference category AOR: adjusted odds ratio, CI: confidence interval

Variables		AOR	95% CI	p-value
Length of stay	Up to three days*			
	More than three days	1.54	0.27-8.86	0.631
Intensive care admission	No*			
	Yes	0.967	0.05-18.64	0.982
Organ support	No*			
	Yes	14.04	0.55-358.32	0.110
Mechanical ventilation	No*			
	Yes	0.910	0.04-20.49	0.952
Ionotropic support	No*			
	Yes	1.10	0.05-24.76	0.952
Dialysis	No*			
	Yes	2.46	0.11-54.40	0.569

## Discussion

Pediatric poisoning represents a prevalent and potentially life-threatening concern, frequently overlooked in households with young children. It accounts for a significant number of visits to ED. In addition to the short-term morbidity, poisoning can lead to death and can have other long-term consequences in children [1,2]. This study aimed to identify common poisoning agents in children, explore risk factors, and determine outcomes.

Most cases of poisoning were observed in the under-three-year age group. Tiwari et al. reviewed pediatric cases of acute poisoning in India and reported that children up to three years of age are the most frequent victims of acute poisoning [17]. A similar trend in the age group distribution is noted by Corlade-Andrei et al. in 2023, Saikia et al. in 2020, Arpitha et al. in 2020, and Khan et al. in 2023, where acute poisoning was most frequently seen in children up to the age of five years [3-5,18].

The number of boys, which accounted for 57.1% of the participants, was higher than the number of girls in the study, indicating a difference in gender representation. This is in concordance with multiple studies where poisoning cases were more frequently observed in male children, especially in the younger age group [6,7,19,20]. Consistent with published literature, all cases of poisoning in this study were accidental and resulted from ingestion of toxic substances [17,18,21]. The natural curiosity and exploratory behavior of boys and mouthing tendencies in young children are supposed to be the main reasons for their frequent involvement in accidental ingestion of toxic substances [17].

The vast majority of cases happened at home. This finding is consistent with reports by Li et al. in 2021 and Ahmed et al. in 2022, where 90.4% and 82% of episodes of acute poisoning in children took place at home, respectively [11,12]. Homes often contain various chemicals and medications that can be hazardous if improperly handled or ingested. The lack of awareness among parents about proper storage and safety measures, such as childproof locks on cabinets, and the natural curiosity of young children who are mostly limited to the home environment, contribute to the higher incidence of poisoning cases within the home environment [11,12]. 

Organophosphorus compounds, followed by household medications and petroleum-based products like kerosene oil, were the most frequent poisoning agents in this study. In a similar study conducted in Nigeria, Areprekumor et al. noted organophosphates and kerosene as the most common agents of poisoning in children [10]. A similar trend has been reported in studies carried out in China and India [22,23]. In contrast, medications are the most frequently ingested poisons in studies from the United States and Brazil [8,24]. The regional variation in the agents of acute poisoning in pediatric patients is well documented, reflecting local practices such as routine use of these chemicals by the local population and inadequate storage methods, which result in easy access for young children.

Most (88.3%) of the children were hospitalized. In a study carried out in Karachi, Pakistan, in 2018-2019, 60.09% of children with acute poisoning were admitted after presenting to a tertiary care hospital [25]. In contrast, lower rates of admission have been reported from South Africa (4.4%), Taiwan (17.2%), and India (40%) [5,6,26]. A small proportion of patients (5.9%, n = 8) required admission to the intensive care unit, which aligns with those reported by Arbaeen et al. and Lamireau et al. [27,28]. Various factors influence the decision to admit these patients, including delays in seeking medical care, the severity of symptoms and signs at presentation, the type of poisoning, the educational level of parents, and the local and regional healthcare infrastructure. The high admission rates in Pakistan may reflect inadequate primary and secondary healthcare infrastructure, ultimately leading to late presentations at tertiary care with established symptoms that require inpatient care.

Most patients (80.9%, n = 110) remained hospitalized for up to three days, with an average stay of 2.8 ± 1.8 days. The relatively shorter hospital stay is a consistent finding in studies on pediatric poisoning, where most cases stayed less than 48 hours, and the mean hospital stay has ranged from 1.8 to 4.8 days [13,14,26]. The duration of the hospital stay primarily depends on the severity of symptoms and signs at presentation and any subsequent complications. A small proportion of patients may require interventions like intensive care admission and organ support that could prolong their hospital stay.

The mortality rate among hospitalized patients was 12.5% (n = 17). Khalid and Rasheed reported a mortality rate of 16.2% in Pakistan [29]. Conversely, the mortality rate for pediatric poisoning cases has varied from 0.3% to 8.7% across several studies conducted outside of Pakistan [10,15,16,18]. Several factors may contribute to the higher mortality rates reported from Pakistan. These include delays in receiving medical care and the subsequent severe symptoms upon presentation, inadequate facilities like non-availability of specific antidotes at most primary and secondary healthcare centers, poor training of primary healthcare personnel in the management of children with poisoning, the lack of awareness and preventive measures among parents, and the widespread presence of highly toxic substances.

Longer hospital stays and the need for intensive care admission are associated with increased mortality. These findings are consistent with those of Uwumiro et al. [30]. These instances likely represent cases with severe symptoms and signs upon admission, necessitating intensive care, organ support, and an extended duration of hospitalization. Due to the relatively small number of fatalities and the link among these risk factors, the length of hospital stay and the requirement for intensive care and organ support were not significant in the multivariate analysis.

This study focuses on acute poisoning cases in children from a region with limited recent data, which is the primary strength of the research, given the well-recognized regional variation in the epidemiology of acute poisoning. The study reviews various aspects of acute poisoning in children and utilizes advanced analysis techniques to mitigate the effects of confounding variables. However, the study's limitations are the retrospective design and small sample size. Given the retrospective nature of the study, its focus on young children who might not have been able to report the agent of poisoning correctly, and the lack of confirmatory testing, the study may have suffered from recall and reporting bias. These factors may have introduced biases in data collection and limited the generalizability of the findings.

## Conclusions

Pediatric poisoning remains a critical public health issue in lower-middle-income countries such as Pakistan. The incidence of acute poisoning was notably high among children under three years of age, with most cases occurring within home settings. Organophosphates, household medications, and petroleum-based products were identified as the predominant poisoning agents. The substantial rates of hospitalization and mortality highlight the severity of the problem. It is imperative to implement educational programs aimed at informing parents about the risks associated with improper storage of medications and toxic substances, as well as the necessity of childproofing their homes. Additionally, enhancing primary and secondary healthcare facilities and access to antidotes for common poisons is essential to minimize delays in obtaining appropriate medical care and improve patient outcomes. These initiatives will also serve as a basis for future research to assess their effectiveness in reducing the incidence of pediatric poisoning.
